# Sex-specific movement ecology of the shortest-lived tetrapod during the mating season

**DOI:** 10.1038/s41598-022-14156-3

**Published:** 2022-06-16

**Authors:** Lennart Hudel, Peter M. Kappeler

**Affiliations:** 1 Department of Sociobiology/Anthropology, University of Göttingen, Kellnerweg 6, 37077 Göttingen, Germany; 2 Behavioral Ecology Unit, German Primate Center, Leibniz Institute of Primate Biology, Kellnerweg 4, 37077 Göttingen, Germany

**Keywords:** Behavioural ecology, Sexual selection

## Abstract

Sex-specific reproductive strategies are shaped by the distribution of potential mates in space and time. Labord’s chameleon (*Furcifer labordi*) from southwestern Madagascar is the shortest-lived tetrapod whose life-time mating opportunities are restricted to a few weeks. Given that these chameleons grow to sexual maturity within about three months and that all individuals die soon after breeding, their mating strategies should be adapted to these temporal constraints. The reproductive tactics of this or any other Malagasy chameleon species have not been studied, however. Radio-tracking and observations of 21 females and 18 males revealed that females exhibit high site fidelity, move small cumulative and linear distances, have low corresponding dispersal ratios and small occurrence distributions. In contrast, males moved larger distances in less predictable fashion, resulting in dispersal ratios and occurrence distributions 7–14 times larger than those of females, and males also had greater ranges of their vertical distribution. Despite synchronous hatching, males exhibited substantial inter-individual variation in body mass and snout-vent length that was significantly greater than in females, but apparently unrelated to their spatial tactics. Females mated with up to 6 individually-known mates, but frequent encounters with unmarked individuals indicate that much higher number of matings may be common, as are damaging fights among males. Thus, unlike perennial chameleons, *F. labordi* males do not seem to maintain and defend territories. Instead, they invest vastly more time and energy into locomotion for their body size than other species. Pronounced variation in key somatic traits may hint at the existence of alternative reproductive tactics, but its causes and consequences require further study. This first preliminary study of the mating system of a Malagasy chameleon indicates that, as in other semelparous tetrapods, accelerated life histories are tied to a mating system with intense contest and scramble competition among males.

## Introduction

One of the dominant axes of diversity in life history traits is the continuum from fast to slow^1,2^, which is characterized by differential allocation of energy to somatic growth, maintenance, and reproduction. Species at the ’fast’ end of this continuum are characterized by rapid growth, quick maturation, high age-specific reproductive effort with many small young, and a short life expectancy, whereas species at the ’slow’ end of the continuum are characterized by the opposite combination of traits. According to life history theory, this diversity is importantly driven by variation in age-specific mortality rates^3,4^. While all major groups of tetrapods contain examples of slow growth, late maturation and extreme longevity^5^, only few taxa are located near the fast end of this life history continuum. Mammals and birds are likely constrained to relatively slow maximum reproductive rates by their obligate parental care, and indeed, only nine species of marsupials are known to have almost-annual life cycles^6^. Similarly, only a few amphibians are known to attain lifespans of less than 2 years^7^. Non-avian reptiles exhibit the greatest variation along the fast-slow axis^8^, with both the longest^9^ and shortest^10^ reported lifespans of terrestrial vertebrates stemming from this group.

Chameleons (Squamata: Chamaeleonidae) are an enigmatic Old World lizard clade encompassing at least 217 species^11^, almost half of which are endemic to Madagascar^12^. They possess numerous unique adaptations to a lifestyle as cryptic, arboreal predators^13^, have elaborate social behavior and signaling^14–17^, and many species show exaggerated sexual size dimorphism as well as conspicuous secondary sexual traits in males^18^. Moreover, chameleons are known for relatively short lifespans compared to other squamates^5,19,20^.

This phenomenon is driven to the extreme by a species from the deciduous dry forests of south-western Madagascar. Labord’s chameleons (*Furcifer labordi*, Grandidier, 1872) spend more of their lifespan as an embryo than as a developed chameleon so that for parts of the year there are only eggs, but no chameleons in the forest; a life history that is unparalleled among tetrapods^10^. Their unique semelparous life history is complemented by a brief reproductive season, in which male-male encounters are more physically intense than in perennial congeners, and females have a longevity advantage similar to that observed in semelparous marsupials^21–23^. Mating behavior appears to be not only determined by male-male competition, because the length of the fleshy rostral appendage of males appears to be evaluated by female mate choice^24^. Observations of vast daily movements (based on unpublished radiotracking data^21^), costly male-male encounters, and mate guarding of solitary females during an extremely short period of reproductive activity raise questions about the optimal male reproductive strategy under these conditions^25^.

Due to their cryptic nature and small to moderate size, studies on the social organization and mating system of chameleons are generally scarce. Radiotelemetry studies have focused primarily on aspects of habitat use, e.g. in invasive East African (*Trioceros* sp.) and Malagasy (*Furcifer* sp.) chameleons in Hawaii and Florida^26–28^ and in South African dwarf chameleons (*Bradypodion* sp.) in semi-urban habitats^29^. Only the mating system of perennial European chameleons (*Chamaeleo* sp.) have been studied in any detail^30–33^. While these studies are informative about the mating systems and spatial strategies of ’conventional’ chameleons, they offer no basis for predicting reproductive strategies in *F. labordi,* however, because the latter’s entire life span is shorter than the age of sexual maturity in many other chameleons, and differences in size among males are much reduced compared to longer-lived species^21^. Moreover, the only currently available information on mating behavior of *F. labordi* is based on observations in staged arena trials^21,24^. We present here the first results of a radiotracking study of wild *F. labordi* during their brief (< 4 weeks) annual mating season, combining spatial and behavioral data for a first characterization of this species’ mating system. Despite its limited duration and sample size resulting from the challenges of finding and following large numbers of males and females in a complex tropical forest habitat, our study provides a first characterization of the key features of the mating system of a Malagasy chameleon.

## Results

### Body size and condition

Female *F. labordi* had a mean body mass of 8.7 g (range 5.5–12.5 g, N = 37) and a mean snout-vent-length of 71.7 mm (range 64.5–80.1 mm, N = 36), while male *F. labordi* had a mean body mass of 16.2 g (range 10.5–24.5 g, N = 24) and a mean snout-vent-length of 92.6 mm (range 75.9–104.9 mm, N = 24). The rostral appendage of male *F. labordi* (N = 22) had a mean height of 5.5 mm (range 4.1–6.7 mm), a mean width of 1.6 mm (range 1.1–2.0 mm) and a mean length of 13 mm (range 9.1–17.2 mm). The mean height of the male cranial casque was 10.9 mm (range 6.8–14.8 mm). Body mass and snout-vent-lengths within male *F. labordi* showed greater variability than within females, with body mass of males detected in the same week differing by up to 110% and snout-vent-length differing by up to 30% (F-test; body mass: F = 0.09, *p* = 0.005, snout-vent-length: F = 0.21, *p* = 0.03). Variance in body condition residuals was also larger among males (range − 0.22 to 0.31) than among females (range − 0.14 to 0.09, F test: F = 0.29, *p* = 0.07).

Body condition and secondary sexual characteristics were not obviously correlated to movement strategies, with the smallest males covering similar 5-day occurrence distributions, cumulative distances and dispersal ratios as the largest males (e.g. M81, initial mass 10.5 g, initial SVL 75.9 mm, five-day-MCP 1860 m^2^, 5-day-cumulative distance 172 m, 5-day-DR 0.21 *versus* M90, initial mass 18.5 g, initial SVL 97.3 mm, 5-day-MCP 1609 m^2^, 5-day-cumulative distance 166 m, 5-day-DR 0.25). In females, body condition and dispersal ratio (DR) appeared to be positively correlated in the 5-day data set (rs: 0.9, *p* = 0.08, n = 5), but were negatively correlated in the full data set (rs: − 0.7, *p* = 0.04, N = 9), indicating that the sample size was too small to obtain biologically meaningful results.

### Ranging

**Figure 1:**
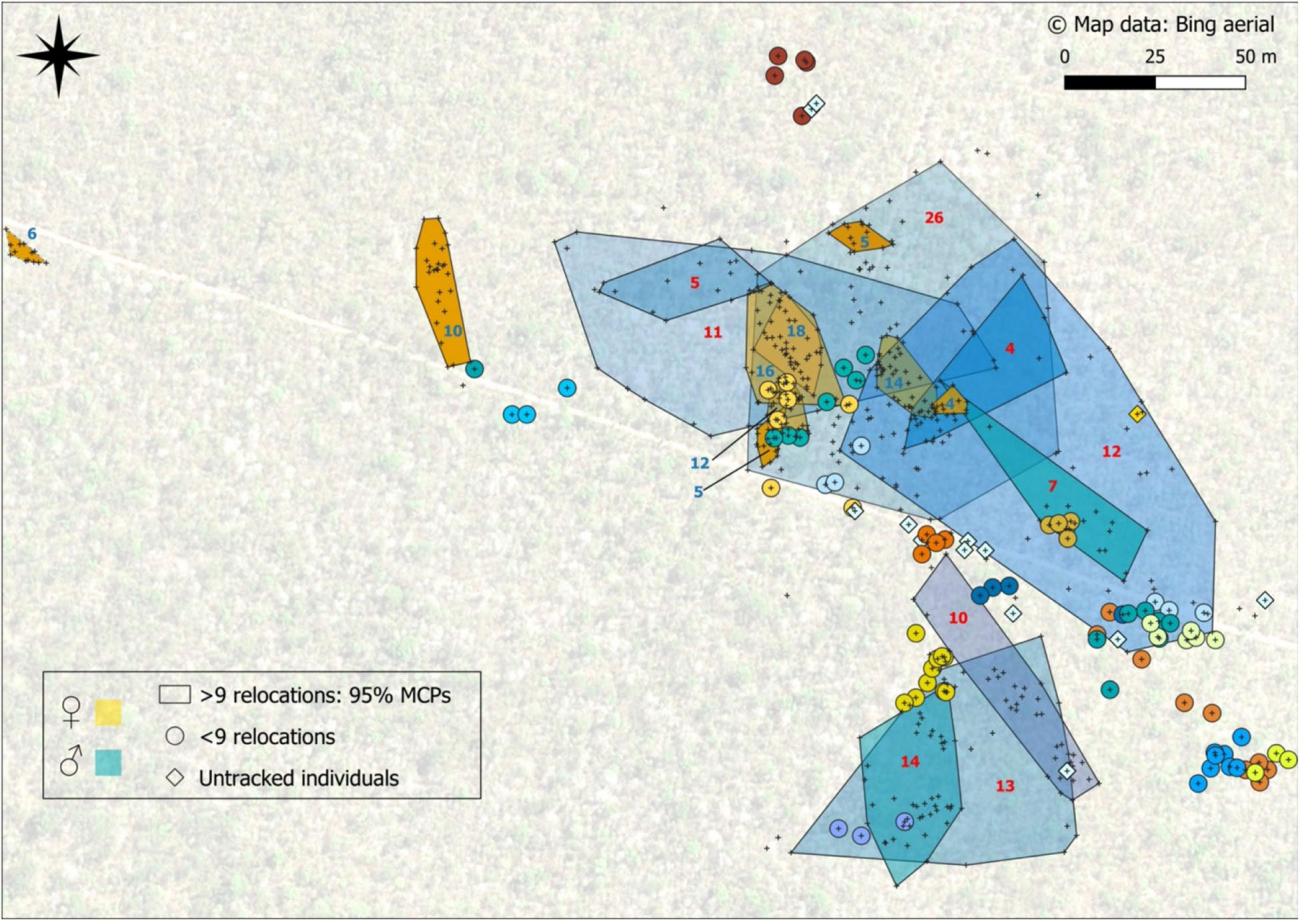
Overview of male (blue) and female (orange) Labord’s chameleons tracked in the study area, showing high local densities and larger occurrence distributions in males. 95% MCPs are shown for all individuals tracked for longer than 3 days, and numbers represent the number of days each individual was tracked. Figure was generated with http://qgis.osgeo.org, using map data licensed by Microsoft Bing Aerial 2021 (https://www.microsoft.com/en-us/maps/product/print-rights).

A total of 39 *Furcifer labordi* (21 females and 18 males) were equipped with radio-transmitters. Of these, 12 females and 9 males lost their transmitters after less than 3 days, i.e., a maximum of 9 relocations. For the remaining 9 females and 9 males, the median number of relocations was 23 (range 10–37) and 26 (range 11–69), respectively, and data from these individuals collected throughout the mating season were used for occurrence distribution and movement trajectory analyses. An additional 14 males and one female were detected in the study area, but roosted too high in the canopy to be captured, illustrating high local densities and indicating that observations on radio-tracked individuals only represent a fraction of individuals and interactions in the habitat at any time (Fig. 1). More precise estimates of the total number of males and females in the study area were not available, however, because of visual constraints and a trade-off between censusing and radiotracking during the extremely short mating season.

### Distances moved

Multiple estimates revealed that males moved longer distances than females. Median 24 h displacement between individual sleeping sites was 7 m (range 1–32) for females (n = 42, 10 individuals) and 14 m (range 2–53) for males (n = 51, 11 individuals), and this difference was statistically significant (Brunner–Munzel test, test statistic 9.98, df = 89, *p* < 0.001). Over their respective tracking duration, females moved a median cumulative distance of 145 m (range 50–192, n = 9), an individual daily average of 9–16 m, whereas males moved a median cumulative distance of 330 m (range 68–760, n = 9), an individual daily average of 23–42 m. Median Euclidean distance (i.e. direct distance from first to last location) was 14 m (range 2.5–30) for females and 64 m (range 6–90) for males.

**Figure 2:**
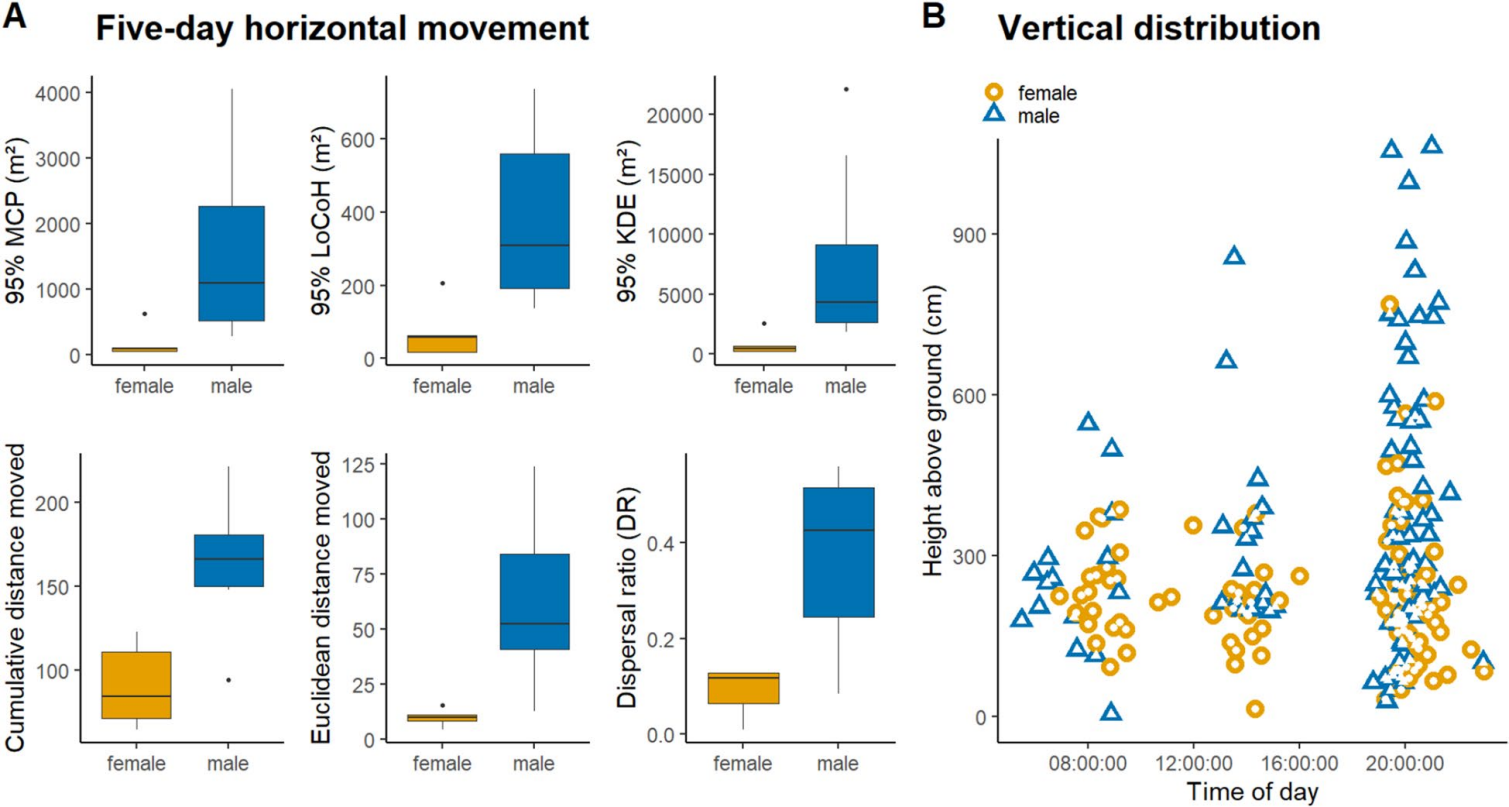
(**A**) Occurrence distribution estimates and other metrics of movement of male (blue) and female (orange) Labord’s chameleons tracked for a time span of 5 consecutive days. Depicted are sex differences in the average areas of the 95% Minimum Convex Polygons (MCP), Local Convex Hull (LoCoH), Kernel Density Estimates (KDE), the cumulative and Euclidian distance moved, as well as the dispersal ratio. (**B**) Vertical distribution of all tracked individuals in the forest canopy.

As these values were derived from unequal tracking periods, we assessed site fidelity for a standardized timespan of 5 days, and calculated a dispersal ratio (DR) by dividing the Euclidean distance through the cumulative distance an individual had travelled in this period. On five consecutive days, females moved a median cumulative distance of 84 m (range 65–123, n = 5), whereas males moved a median cumulative distance of 158 m (range 92–221, n = 8). Median Euclidean distance away from the initial point of encounter over a 5-day-period was 10 m (range 5–16) for females and 53 m (range 13–124) for males. The median dispersal ratio resulting from these respective values was 0.12 ± 0.01 (range 0.06–0.13) for females and 0.43 ± 0.06 (range 0.13–0.56) for males. Both estimates and the dispersal ratio were significantly larger in males than in females (permuted Brunner–Munzel test, cumulative distance: sample estimate 0.95, *p* = 0.005; Euclidean distance: sample estimate 0.975, *p* = 0.003; dispersal ratio: sample estimate 0.925, *p* = 0.009, Fig. 2A).

### Occurrence distribution estimates

All occurrence distribution estimates suggest that males covered larger areas than females. Median MCP areas (95% level of Minimum Convex Polygon) were 144 m^2^ for females (n = 9) and 1037 m^2^ for males (n = 9), and the difference between males and females was statistically significant (permuted Brunner–Munzel test, sample estimate 0.99, *p* < 0.001, Fig. 2A). Median MCP core areas (50% level) were 58 m^2^ for females and 248 m^2^ for males, and male core areas were also significantly larger than those of females (permuted Brunner–Munzel test, sample estimate 1, *p* < 0.001). While 95% MCP area and the number of relocations per individual were correlated (Kendall’s tau: 0.35, z = 2.01, *p* = 0.044), standardized five-day MCP areas (95% level) were also significantly larger for males (median 1097 m^2^, n = 8, range 286–4050) than for females (median 97 m^2^, n = 5, range 44–634, permuted Brunner–Munzel test, sample estimate 0.9, *p* = 0.018). Of individuals in physical proximity, i.e. having a 95% MCP area overlap greater than 0, male MCPs overlapped with a median of 5 (range 1–11) other individuals, while female MCPs overlapped with a median of 3.5 (range 1–5) other individuals. Of 6 males and 6 females with adjacent or overlapping MCPs, mean distances between centroids of individual locations were 34 m for female pairs (range 5–62, n = 42), 59 m for male pairs (range 9–122, n = 32) and 50 m for male–female pairs (range 5–95, n = 42).

Median LoCoH areas (95% level; Local Convex Hull) were 62 m^2^ for females and 491 m^2^ for males. 95% LoCoH area and the number of relocations per individual were not significantly correlated (Kendall’s tau: 0.31, z = 1.78, *p* = 0.075). The difference in 95% LoCoH estimates between males and females was significant (permuted Brunner–Munzel test, sample estimate 0.94, *p* < 0.001, Fig. 2A). Median LoCoH core areas (50% level) were 10 m^2^ for females and 46 m^2^ for males. This difference in core area between males and females was significant (permuted Brunner–Munzel test, sample estimate 0.95, *p* < 0.001). Standardized five-day LoCoH OD areas (95% level) were also significantly larger for males (median 309 m^2^, range 137–736) than for females (median 59 m^2^, range 18–188), permuted Brunner–Munzel test, sample estimate 0.925, *p* = 0.011). The LoCoH estimates were also affected by sample size, however, and should therefore be interpreted with caution.

Median KDE areas (95% level; Kernel Density Estimate) were 488 m^2^ for females and 6694 m^2^ for males. 95% KDE area and the number of relocations per individual were not correlated (Kendall’s tau: 0.26, z = 1.48, *p* = 0.139), and the difference in 95% KDE area between males and females was significant (permuted Brunner–Munzel test, sample estimate 1, *p* < 0.001, Fig. 2A). Median KDE core areas (50% level) were 132 m^2^ for females and 1457 m^2^ for males, and this difference was statistically significant (permuted Brunner–Munzel test, sample estimate 1, *p* < 0.001). Standardized five-day KDE OD areas (95%) were also significantly larger in males (median 4399 m^2^, range 1821–22,102) than in females (median: 441 m^2^, range 180–2570, permuted Brunner–Munzel test, sample estimate 0.95, *p* = 0.006).

Median roosting height of females was 205 cm (range 14–769 cm), while males had a median roosting height of 270 cm above ground (range 4–1062 cm). Among the 18 tracked individuals for which multiple measurements were available, individual range of vertical distribution, i.e. range from lowest to highest recorded height above ground, was higher in males (median 467 cm, range 170–1014, N = 9) than in females (median 250 cm, range 87–565, N = 9; Fig. 2B), indicating that males are more mobile in the third dimension as well.

### Mating system

Due to low transmitter retention, difficult observation conditions and an unknown number of additional individuals present in the study area, the following accounts of mating behavior should be considered as preliminary*.* One male (M81) traversed the areas utilized by 10 radio-tracked females, for 7 of which occurrence distribution estimates are available and 3 of which were located 4–7 times. It was observed courting or mate guarding 6 of these females, and engaged in altercations with other known males pursuing the same females at least 3 times. The occurrence distributions of the females sequentially mated by this male during this period of ca. 3 weeks were traversed by 10 known radio-tracked males during this period, and an additional 6 unmarked males were observed in the area. Four other tracked males and one unmarked male either mate guarded or stayed close to the same females as M81 during the same period, and encounters between males occurred at least 3 times. All males showed erratic movement trajectories with both rapid ’bursts’ interspersed by longer periods with little or no movement. These observations suggests that other male *F. labordi*, even though their interactions with known and unknown conspecifics in the habitat were often impossible to detect, pursued a similar strategy of rapid sequential mating attempts.

Different males were repeatedly tracked at relatively small distances to each other (< 10 m), and also always in close vicinity to at least one known female’s location. It is unclear, however, whether fights between these individuals took place, whether they were aware of each other (and potentially additional, non-tracked males), and whether some of the large distances traversed by males between tracking locations were due to displacements by other males. In two instances, two different males slept in direct vicinity of each other (< 3 m and < 5 m distance, respectively) and close to a nearby female. Several tracked males showed bite marks at the tail base or abrasions at the rostral appendage and jaw, likely a result of ’jawlocking’ and biting during combat.

Of the 21 radio-tracked females, seven deposited eggs during the period of tracking, and three clutches containing 6–8 eggs were recovered. However, because the timing of fertilization could not be determined for all females, it was impossible to analyze female ranging behavior as a function of reproductive status. Six females entered abandoned terrestrial ant burrows (of the genus *Camponotus*) for egg deposition. Of those, one deceased within 12, and another one within ca. 48 h after egg deposition, highlighting the large energetic investment into reproduction in this species.

## Discussion

By radiotracking male and female *F. labordi* during their short annual reproductive activity, this study provided first baseline data on the sex-biased movement ecology and underlying mating strategies of the shortest-lived tetrapod. Our data indicated that females exhibited high site fidelity, with low cumulative and linear distances moved, and corresponding low dispersal ratios and small occurrence distributions. In contrast, males moved larger distances in less predictable fashion, resulting in higher dispersal ratios and occurrence distributions 7–14 times larger than those of females. Consequently, their occurrence distributions encompassed those of several females, with males apparently searching for and visiting one female after another, interrupted by agonistic interactions and displacement when encountering male rivals.

Low site fidelity, small distances between individual center points, and strong overlap of occurrence distributions in males suggest that, unlike in perennial European chameleons^33^, *F. labordi* males do not maintain and defend territories. This notion is further supported by anecdotal evidence: one male traversed 10 and mate-guarded or courted at least 6 known females within a period of 21 days, and it encountered other radio-tracked males attempting to breed with the same females at least 3 times. As additional males were regularly observed in this area, and as further individuals of both sexes likely remained completely undetected during the duration of the study, the true number of females courted per male, courting males per female, and of agonistic male-male encounters is likely even higher. In combination, these observations suggests that *F. labordi* has a polygynandrous mating system with pronounced contest and scramble competition among males. Below, we will compare the movement ecology of *F. labordi* to what is known from longer-lived relatives, and will discuss its association with life-history traits, reproductive strategies, and the resulting mating system.

### Movement ecology

Occurrence distributions estimated in the present study are larger than estimates reported for larger and longer-lived chameleon species^33,34^. Male European chameleons (*C. chamaeleon*) utilized 100% MCPs ranging from 40 to 719 m^2^ during their reproductive season^33^ (Cuadrado 2001), averaging ca. 1/10th of 95% MCPs found for male *F. labordi*. It is therefore apparent that male *F. labordi* cover vastly larger areas than the only other chameleon species for which movement data during the reproductive season exist. Considering the smaller body size of *F. labordi*, males of this species therefore appear to invest proportionally more time and energy into locomotion than their European relatives.

Movement strategies of *F. labordi* clearly differ between the sexes and provide insights into their mating system. Male-biased movement metrics have been associated with polygynous mating systems in mammals^35^. The estimates of movement generated here (combined with the observations on the mating behavior of *F. labordi*) are strikingly congruent with this notion, perhaps with the exception that female defense appears to be neither beneficial nor feasible in this extremely short-lived species. Nonetheless, we cannot exclude the possibility with the presently available preliminary data that some males patrolled an area that contained multiple females whereas others, notably smaller males, used a sneaker strategy that was also associated with high locomotor activity.

Male-biased increased movement as a mate-finding strategy has been suggested for several vertebrate species^36–38^. For example, males with larger home ranges had access to more potential mates in speckled rattlesnakes (*Crotalus mitchellii*), where strong competition arises from the clumped spatiotemporal distribution of receptive females^38^. Investment in presumably costly mate searching appears to be the driver of male-biased movement in *F. labordi* as well. The observed variation in movement estimates among different males may therefore be indicative of the number of females encountered. Because movement estimates were not linked to metrics of body condition or secondary sexual characters, it remains unclear why some males moved less than others, however, without displaying obvious territoriality or mate defense. Thus, there is variation in male movement ecology that remains to be explained.

Movement of female *F. labordi* was much more confined than that of males, indicating that roaming is less beneficial for females. High site fidelity might instead be beneficial when resources are locally abundant and do not exhibit spatio-temporal variation, a scenario that applies to the deciduous forest habitat of these chameleons only during the short rainy season. As feeding resources and small-scale thermal gradients appeared to be at no shortage (e.g. in almost 50% of direct observations, females were feeding), females might not need to move large horizontal distances, and can apparently sustain themselves in small and even overlapping home ranges. Indeed, large movements were exclusively observed in gravid females, apparently searching for suitable egg deposition sites, but the limited available data did not permit a formal analysis of the effects of female reproductive state on their ranging behavior because the exact dates of mating were not known for most females. The divergent mate acquisition strategies of males and females appear therefore not to be driven by limited feeding resources and structural or thermal requirements. Instead, males engage in movement to increase their number of encounters with females, at the cost of increased predation risk and encounters with competitors. Females, in contrast, might face no limiting resource other than suitable egg deposition sites, rendering them more philopatric.

### Life history traits

Given the prevalence of male-male combat and female choice reported for this species^21,24^, the sex differences in secondary sexual characters observed here are to be expected. However, inter-individual variation of up to 110% in body mass and 30% in snout-vent-length among individuals present at the same time was unexpected, considering the synchronous hatching of *F. labordi*. With the short period of female receptivity being the limiting factor, males growing and maturing at the fastest rate should enjoy the greatest mating success, if male size confers reproductive advantages. The observed pronounced variation in male body condition might therefore be the result of a male arms race, but it remains unknown which factors allow or limit growth to particularly large or small sizes, respectively. There is experimental support for the importance of male body size in both intra- and intersexual selection^21^, but it remains unknown how frequent and under which circumstances male-male and male–female encounters occur under wild conditions, and which fitness consequences body condition and secondary sexual characteristics have for individual reproductive success. Besides, 35% of experimental male-male altercations were won by the smaller competitor^21^, highlighting that additional factors play a role in intrasexual selection.

Small males ought to have a substantial disadvantage concerning mating opportunities and success^32^, unless they follow a sneaker strategy. However, data from the single male for which a detailed account of mate guarding and mating attempts could be compiled contradicts this notion: it was the smallest of all radio-tracked males in terms of both SVL and body mass when first equipped with a transmitter (first record: 76 mm, 10.5 g), and even though it grew during the study (last record: 84.2 mm SVL, 11.5 g), it never reached nearly the size of some other males in the same area at the same time (e.g. last records of 103.5 mm SVL, 23.5 g or 104.9 mm, 20 g). While the outcome of male-male altercations and mating attempts of this male remain unknown, it pursued the same movement strategy as vastly larger males. Additional data are now required to determine whether males of different size pursue alternative male reproductive tactics^39^.

### Mating system

Because most previous chameleon studies of processes involved in accessing and choosing mates have been conducted with animals in permanent or temporary captivity^14–17,24^, empirical data on chameleon mating systems are scarce^30–34,40,41^. While it has been suggested that they possess a wide range of different mating systems^40^, the clade is generally assumed to be polygamous in the sense that "(m)ales may mate with more than one female and females may mate with different males during the same or different ovarian cycles"^40^ when given the opportunity. Whether females mate with one or multiple males within one ovarian cycle is likely dependent on encounter rates and male monopolization potential. Studies in captivity indicated that the dynamics of male color change may impact female mate choice^42^ and predict the outcome of male-male agonistic interactions^43^, but the relative importance of these processes in shaping the mating system of wild (panther) chameleons remains unknown. Our study is therefore the first to contribute observations on the mating system of a wild population of Malagasy chameleons.

Compared to perennial chameleons, in which some males are able to monopolize the most fecund females for up to 46 days^32^, male monopolization potential in *F. labordi* appears to be much reduced, but additional observations of matings are required to better assess the strategies males use to defend their reproductive success. Some populations of Canadian garter snakes (*Thamnophis sirtalis*) also have just a few weeks to mate after larger numbers of spatially clumped females emerge from communal overwintering dens. They mate promiscuously but males with better body condition are more successful and use mating plugs rather than mate guarding or extended mating to secure their reproductive success^44^. More comparative data on male reproductive tactics in other reptilian taxa with short breeding periods are required for a more detailed appraisal of the available options.

While clutch size is strongly correlated to female body size in chameleons^45^, it is unlikely that variation in female *F. labordi* fecundity is similarly larger as in the larger perennial congeners, for which estimates of 10–30 (*F*. cf. *nicosiai*) and an average of 42 eggs (*F. oustaleti*) have been reported^23^. Three females during this study had clutches containing 6, 7 and 8 eggs respectively, indicating some variation in fecundity among female *F. labordi*, but we found no evidence for a decreasing size-rank-order of male mate guarding, as observed in perennial European chameleons^33^. As female squamates are able to store sperm internally, and therefore can control the timing of fertilization and possibly paternity^46,47^, male mating success does not guarantee fertilization success. It is therefore in the interest of males to maximize both copulation duration per female and the number of mates. Since the vast majority of female *F. labordi* produce only a single clutch during their lives^23^, mating polyandrously might reduce the risk of producing unfertilized eggs and improve genetic diversity of their offspring^48^.

In contrast, males can generally increase their reproductive success by mating with additional females. As only about 5% of females in *F. labordi* experience a second ovarian cycle^23^, the operational sex ratio changes drastically as the breeding season advances, which may explain the parallel increase in male agonism^49^. With increasingly male-biased operational sex ratios, levels of polyandrous matings and multiple paternity ought to increase as well^50^, but future studies are required to test these predictions. Larger sample sized would also permit studies of the effects of sex ratio dynamics on male ranging behavior.

## Conclusions

This work represents the first study of the mating system of a Malagasy chameleon, and provides first insight into the movement ecology and mating system of the shortest-lived tetrapod. Perhaps due to low variation in female fecundity, and both spatial and temporal factors that impede the formation and defense of stable territories, there appears to be neither an incentive nor an opportunity for male *F. labordi* to monopolize receptive females. Observed variation in movement patterns, body size and secondary sexual characters were not indicative of alternative reproductive strategies among males, but additional studies of individually known males across their entire life span will be required to identify potential causes and behavioral consequences of striking variation in male body size. Sex differences in movements in all 3 dimensions indicate the prevalence of a polygynandrous mating system, characterized by pronounced male contest and scramble competition and female polyandry. The mating system of *F. labordi* therefore resembles that of other semelparous tetrapods^51^, suggesting that accelerated life histories are tied to particular mating systems.

## Methods

### Study site

The study was conducted in Kirindy Forest (44°39′E, 20°03′S, ca. 30–60 m asl), which is located ca. 60 km north of the coastal town of Morondava and ca. 20 km inland from the Mozambique channel in the central Menabe region of western Madagascar. The climate in this area is characterized by a short, hot and wet austral summer between November and March, and a cooler austral winter with little or no rainfall^52^. The study area, locally known as CS7, is a 1 km^2^ forest block intersected by the Kirindy River and covered by a grid system of foot trails 25 m apart that allow orientation and movement through the dense forest.

### Radio-tracking of *Furcifer labordi*

Between 23 January 2020 and 09 March 2020, male and female *F. labordi* were searched for and equipped with small radio-transmitters (BD-2, 0.64 g, 151 MHz, Holohil Systems Ltd.), using a cyanoacrylate adhesive (UHU ’superglue flex gel’, Bolton adhesives). The transmitter weighed less than 7.4% of the average female mass and less than 4% of the average male mass. The study had to be terminated due to a Covid-related evacuation. Individuals were searched for along the grid system between 18:30 and 22:00 h, using a Fenix HL60R LED headlamp. Upon detection, their roosting height above ground was measured (using a Bosch PLR50 laser rangefinder), as well as their body temperature at the center of the lateral body surface (using a Laserliner ThermoSpot infrared thermometer). Chameleons were then captured to measure their snout-vent-length (SVL) and, in males, two secondary sexual characters to the nearest 0.1 mm with digital calipers: The height, width and length of the rostral appendage as well as the height of the cranial casque. Body mass was then measured using a Pesola spring balance (60 g, d = 0.5 g).

Chameleons were immobilized in a piece of soft cloth and the transmitter was attached above the left or right hip of the animal, taking advantage of their dorso-laterally flattened body. Curing of the adhesive took between 4 and 10 min. Each transmitter carried the two-digit serial number that was often visible with binoculars and also served as the animal ID. At the initial encounter and during all subsequent visual encounters with tracked individuals, a photograph of the animal was taken. After controlling the correct fit of the transmitters, the animals were released onto the same branch they were collected from, and a GPS coordinate was taken, using a Garmin eTrex 20x. To facilitate subsequent tracking of the animals, the location was marked using a colored flag.

Chameleons were tracked using a Telonics TR-4 receiver and F150-151-3FB antenna. Each individual was located three times per day: between 07:00 and 10:00 h, between 12:00 and 15:00 h, and between 18:30 and 21:30 h, covering both diurnal activity patterns and nocturnal roosting site choice. Conventional triangulation methods were used, facilitated by the grid system. Upon visual detection of an individual, its height above ground and body temperature were measured as described above. Focal individuals were observed for 2–5 min, and their current activity (sleeping, resting, feeding, moving, interaction with conspecifics, i.e., mate guarding, courtship or agonistic behavior, other) was noted. The GPS coordinate was then taken, and the location of the animal was marked. Due to the dense and sometimes impenetrable undergrowth and the rich foliage during rainy season, it was not always possible to establish visual contact with the tracked individual, especially when the animals were active high in the canopy. In these cases, the GPS coordinate of the shrub or tree with the strongest signal was taken and marked.

### Data analyses

Data storage and exploration was conducted in Excel v16.0. Statistical analyses were conducted in R v4.0.2^53^ using RStudio v1.3.959^54^. The packages *plyr*^55^, *dplyr*^56^ and *lubridate*^57^ were used for data wrangling, and the packages *ggplot2*^58^ and *cowplot*^59^ were used for the creation of graphs. The packages *psych*^60^, *modelr*^61^ and *car*^62^ were used to perform descriptive statistics. The package *brunnermunzel*^63^ was used to perform nonparametric tests of stochastic equality for two samples with unequal variances in non-normally distributed data. We used the residuals of the linear regression between (log-transformed) body mass and SVL to operationalize body condition. Spearman’s rank correlation tests were conducted to test for relationships between standardized movement metrics and measurements of body size, body condition, and measurements of secondary sexual characteristics. To assess the areas utilized by radio-tracked individuals, relocation data were used to estimate occurrence distributions. Terminology in space-use studies has often been ambiguous^64^, and this term was proposed recently^65^, replacing the more commonly used term home range^66^ when it is not applicable.

The package *amt*^67^ was used for the calculation of minimum convex polygon (MCP), local convex hull (k-LoCoH) and kernel-density-estimation (KDE) estimates of occurrence distributions, and for the analysis of simplified movement trajectories. Other geospatial analyses and the compilation of maps were conducted in QGIS v3.10.0^68^. Using the MCP method, the smallest polygon including all GPS points with interior angles less than 180 degrees was constructed. To exclude outliers, MCP estimates are commonly truncated to include only 95% of all available relocations for one animal. In addition, 50% MCPs were calculated to identify core areas of utilization. While MCPs are the classical and most intuitive method for estimating home range size, they have the tendency to include areas that are not utilized by the animal at all, and by default, their edges can only be of convex shape. Some of these limitations are avoided using the k-LoCoH method^69^, where MCPs, here called local convex hulls, are constructed for local subsets of each point in the dataset and its nearest neighbors. The resulting hulls are then merged from smallest (containing most locations) to largest (containing least locations) and can then be divided into isopleths containing a certain percentage of the whole data set. Unlike MCPs, LoCoHs allow for concavity and ’holes’ in the estimated area. To compare the two methods, LoCoH estimates were also constructed at a 95% level, and, as the method is particularly suited to assess core areas, at a level of 50%. The KDE method^70^ is a modelling approach that fits a probabilistic function to each data point, resulting in a surface of continuous probability density for the relocations of the animal. Based on this surface, isopleth contours containing home range and core area are calculated. Here, reference bandwidth selection for the smoothing of the kernels was used, and the 95% isopleth was considered the occurrence distribution, while the 50% contour was considered the core area. Finally, the dispersal ratio was calculated as described previously^71^.

### Ethics approval

This study adhered to the Guidelines for the Treatment of Animals in Behavioral Research and Teaching (Animal Behaviour 2020) and the legal requirements of the country (Madagascar) in which the work was carried out. The protocol for this research was approved by the Malagasy Ministry of the Environment, Water, and Forests (296/19/MEDD/SG/DGEF/DGRNE).

### Consent for publication

The authors consent to the publication of this manuscript in Nature Scientific Reports.

## Data Availability

The datasets used and/or analysed during the current study available from the corresponding author on reasonable request.

## Code availability

The code used during the current study available from the corresponding author on reasonable request.
